# The AP2/ERF Gene Family in Camphor Tree: Structure, Evolution, and Transcriptional Response to Epicoccum Infection

**DOI:** 10.3390/plants14172694

**Published:** 2025-08-28

**Authors:** Jiexi Hou, Jinrui He, Yiran Liu, Zhufei Xiao, Haiyan Zhang, Changlong Xiao, Rong Zeng, Hongjian Wan

**Affiliations:** 1 Jiangxi Provincial Engineering Research Center for Seed-Breeding and Utilization of Camphor Trees, Nanchang 330099, China; jxhou@cctnit.com (J.H.); zufei007@163.com (Z.X.); zhanghaiyansw03@163.com (H.Z.); clxiao@nit.edu.cn (C.X.); zrong6102@163.com (R.Z.); 2 School of Soil and Water Conservation, Jiangxi University of Water Resources and Electric Power, Nanchang 330099, China; he_jr2001@126.com (J.H.); lyrzlz@163.com (Y.L.); 3 State Key Laboratory for Quality and Safety of Agro-Products, Institute of Vegetables, Key Laboratory of Vegetable Germplasm Innovation and Quality Breeding in the Province, Zhejiang Academy of Agricultural Sciences, Hangzhou 310021, China

**Keywords:** AP2/ERF transcription factors, camphor tree, genome-wide analysis, growth–defense trade-off

## Abstract

The AP2/ERF transcription factor family plays pivotal roles in plant growth, stress responses, and defense mechanisms, yet its diversity in camphor trees remains underexplored. This study identified 154 AP2/ERF genes in the *Camphora officinarum* genome, with over 80% belonging to the ERF subfamily, a distribution consistent with other angiosperms. Synteny analysis revealed that tandem and segmental duplications were key drivers of family expansion, suggesting adaptive diversification under ecological pressures. Structural analysis showed that the majority of ERF/RAV subfamily genes possess a single-exon structure, whereas AP2 subfamily genes display muti-exon structures, indicating divergent evolutionary trajectories and potential functional versatility via alternative splicing. Promoter analyses detected numerous hormone- and stress-responsive elements, linking these genes to abscisic acid, auxin, gibberellin signaling, and pathogen defense. Further expression profiling during stem development showed that approximately 60% of CoAP2/ERF genes were constitutively expressed across 17 expression trends, suggesting roles in basal development and stage-specific processes (e.g., lignification). Under *Epicoccum poaceicola* infection, 23 CoAP2/ERF genes were differentially expressed. Among them, upregulated ERF homologs related to *RAP2.2/2.3* suggested roles in hypoxia and antimicrobial responses, while downregulation of *ERF5* homologs indicated a growth–defense trade-off, whereby developmental processes are suppressed to prioritize pathogen resistance. Overall, this study deciphers the genomic architecture and structural diversity of CoAP2/ERF genes, along with expression dynamics of these genes in development and biotic stress adaptation of camphor trees. These findings provide critical insights into transcriptional regulation of development and stress responses in camphor trees and establish a theoretical basis for molecular breeding and biotechnological strategies aimed at improving stress resilience in woody plants.

## 1. Introduction

*Camphora officinarum* (camphor tree) is an indigenous evergreen species naturally distributed across China and widely cultivated throughout Asian countries. The tree is rich in essential oils, primarily composed of linalool, borneol, citral, and other bioactive fragrant compounds. These components are extensively used in pharmaceutical, cosmetic, and food industries, holding significant economic value, which in turn drives the commercial cultivation of camphor trees for essential oil production [1]. It is well known that essential oils derived from camphor trees exhibit inhibitory properties against plant pathogens, implying that this tree species could be more resilient to diseases and insect invasions. However, under the influence of urbanization and climate change, not only previously existed but also newly emerged diseases, such as anthracnose caused by *Colletotrichum* spp., witches’ broom disease caused by *Candidatus* Phytoplasma asteris, leaf spot disease caused by *Epicoccum poaceicola*, and root rot caused by *Phytopythium vexans*, were frequently observed in camphor tree cultivars, causing a significant loss in biomass and even eventual tree death [2,3,4,5]. Developing stress-tolerant cultivars ensures consistent oil yield and secures supply chains for camphor trees; it is therefore urgent to investigate the molecular basis of disease resistance in these tree species.

The AP2/ERF (APETALA2/Ethylene Responsive Factor) gene family represents one of the largest transcription factor families in plants. These genes are distinguished by the presence of one or two conserved AP2/ERF DNA-binding domain(s), a 60–70 amino acid motif that facilitates binding to specific DNA sequences [6]. With advancements in genome sequencing, genome-scale analyses of AP2/ERF transcription factor family have been conducted in many plant species, including Arabidopsis (*Arabidopsis thaliana*), rice (*Oryza sativa*), grape (*Vitis vinifera*), tomato (*Solanum lycopersicum*), ginger (*Zingiber officinale*), and aspen (*Populus trichocarpa*) [7,8,9,10,11]. These studies have provided insights into the evolutionary history of this transcription factor family and aided in the subclassification of its genes.

Initially, Riechmann et al. [12] classified AP2/ERF members into three subfamilies, including AP2, ERF, and RAV (Related to ABI3/VP1), based on their sequence similarities. Sakuma et al. [13] refined this classification by dividing the ERF family into two subfamilies, which are the ethylene responsive ERF and ethylene non-responsive DREB (Dehydration Responsive Element Binding) subfamilies. Additionally, a Soloist subfamily was introduced to accommodate a few unclassified factors within the larger AP2/ERF family [13]. Further refinement was achieved through phylogenetic and structural analyses, which included examining exon/intron patterns and protein motifs. This led to an alternative classification system that classified AP2/ERF members into four main subfamilies, including the AP2/ERF domain-containing AP2 subfamily, ERF (incorporating DREB), RAV, and Soloist subfamilies. In this scheme, the ERF subfamily was divided into 12 groups, labeled from I to X, along with VI-L (VI-like) and Xb-L (Xb-like) [7].

Refinement made in classification schemes, in turn, has significantly facilitated the functional annotation of family members. Genes from AP2 subfamily are pivotal in regulating developmental processes such as vegetative growth, floral organ identity, and seed development [14,15,16]. ERFs are particularly significant in modulating responses to hormonal signals such as ethylene, which is essential for metabolic regulation, plant growth and development, and responses to biotic and environmental stresses [17,18,19,20]. In addition, ERFs act at the convergence points of multiple signaling pathways, facilitating crosstalk between plant hormones such as ethylene, jasmonate, and abscisic acid, as well as redox signaling in plant responses to abiotic stresses [21]. Meanwhile, RAV genes primarily participate in leaf senescence and also play a role in mediating both biotic and abiotic stress responses [22,23].

In a recent study of camphor tree leaves using single-cell RNA sequencing, members of the ERF subfamily were observed to be differentially expressed among various cell types, suggesting their potential roles in modulating processes such as developmental transitions, secondary metabolism, and stress responses [24]. However, the evolutionary dynamics, structural features, and regulatory roles of AP2/ERF genes in camphor trees remain largely unknown. In particular, it is unclear how CoAP2/ERF genes modulate fundamental development processes and defense responses in this ecologically and economically significant tree species. *E. poaceicola*, a necrotrophic fungal pathogen, causes leaf spot disease and tissue necrosis in camphor trees, often leading to significant biomass loss. Understanding how CoAP2/ERF genes respond to this pathogen is therefore essential for uncovering the molecular basis of camphor tree defense. To address these gaps, this study has two primary aims: (1) to conduct a genome-wide identification of CoAP2/ERF gene family members in camphor trees using the recently released high-quality *C. officinarum* reference genome and (2) to investigate the expression profiles of these genes across stem development and under *E. poaceicola* infection, focusing on their dual roles in development and biotic stress adaptation, as well as the potential growth–defense trade-off. The results of this study provide not only a comprehensive overview of the AP2/ERF gene family but also valuable insights into genetic modification and molecular breeding strategies aimed at developing camphor trees with enhanced defense capabilities.

## 2. Results

### 2.1. Identification and Classification of AP2/ERF Transcription Factors in C. officinarum

A genome-wide Hidden Markov Model (HMM) search identified 154 AP2/ERF candidates. Based on conserved domain composition and functional annotation against NCBI resources, they were classified into three subfamilies: AP2 (23), ERF (128), and RAV (3). Specifically, 22 proteins with two AP2 domains were assigned to the AP2 subfamily, 127 proteins with a single AP2/ERF domain to the ERF subfamily, and three proteins containing one AP2/ERF domain plus a B3 domain to the RAV subfamily (Table 1).

**Figure 1:**
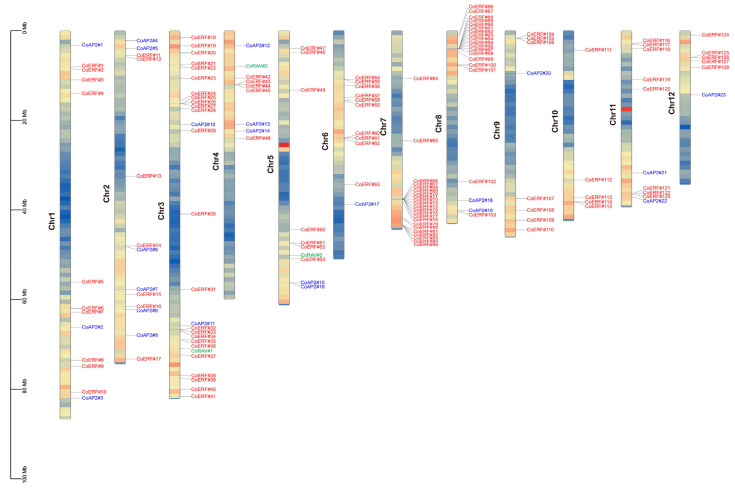
Chromosomal locations of CoAP2/ERF family members. Genes were renamed according to their annotation and chromosome locations. Color indicates gene density: red for the most densely populated regions, blue for the least.

All genes were mapped to the 12 chromosomes of the camphor tree genome and renamed according to chromosomal location as *CoAP2#1–#23*, *CoERF#1–#128*, and *CoRAV#1–#3* (Supplementary Table S1). Chromosome-level mapping shows that AP2/ERF loci are more frequently located toward chromosomal ends, with the largest counts on chromosomes 3, 7, and 8 (27, 22, and 20 genes, respectively). We observed locally enriched regions of AP2/ERF genes on several chromosomes—most notably on chromosomes 7 and 8—based on visual inspection of gene positions (Figure 1). These observations are descriptive only; no statistical testing of non-random distribution was performed in this study. For consistent annotation, we refer to a “tandem/clustered” arrangement when adjacent AP2/ERF genes are separated by ≤10 non-family genes or lie within ≤200 kb, without implying statistical enrichment.

**Figure 2:**
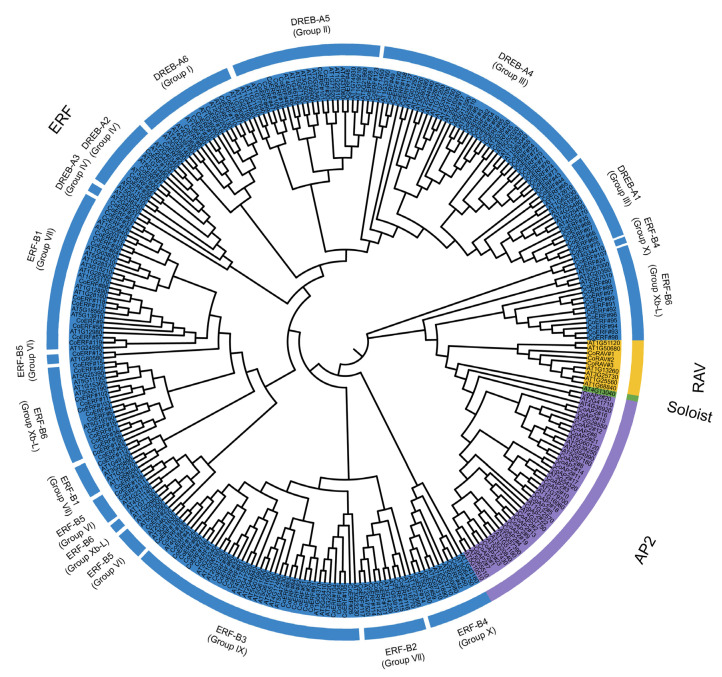
Phylogenetic analysis of AP2/ERF transcription factors from camphor tree and Arabidopsis. Different colors indicated genes from various subfamilies. Only ERF, AP2, and RAV genes (with the prefix “Co”) were observed in the camphor tree.

To explore the evolutionary relationships among these genes and further refine their classification, phylogenetic analysis was conducted alongside 147 AP2/ERF family members from Arabidopsis [7]. This classification scheme aligned well with our initial classification. It not only confirmed the absence of any members in the Soloist subfamily but also assigned *CoAP2#20*, which contains one AP2/ERF domain, and *CoERF#97*, which contains two AP2/ERF domains, to the AP2 and ERF subfamilies, respectively. Additionally, genes from the ERF subfamily can be further divided into groups I-VII and Xb-L, according to classifications made in Arabidopsis (Figure 2).

### 2.2. Gene Structure and Motif Analysis of AP2/ERF Genes in C. officinarum

**Figure 3:**
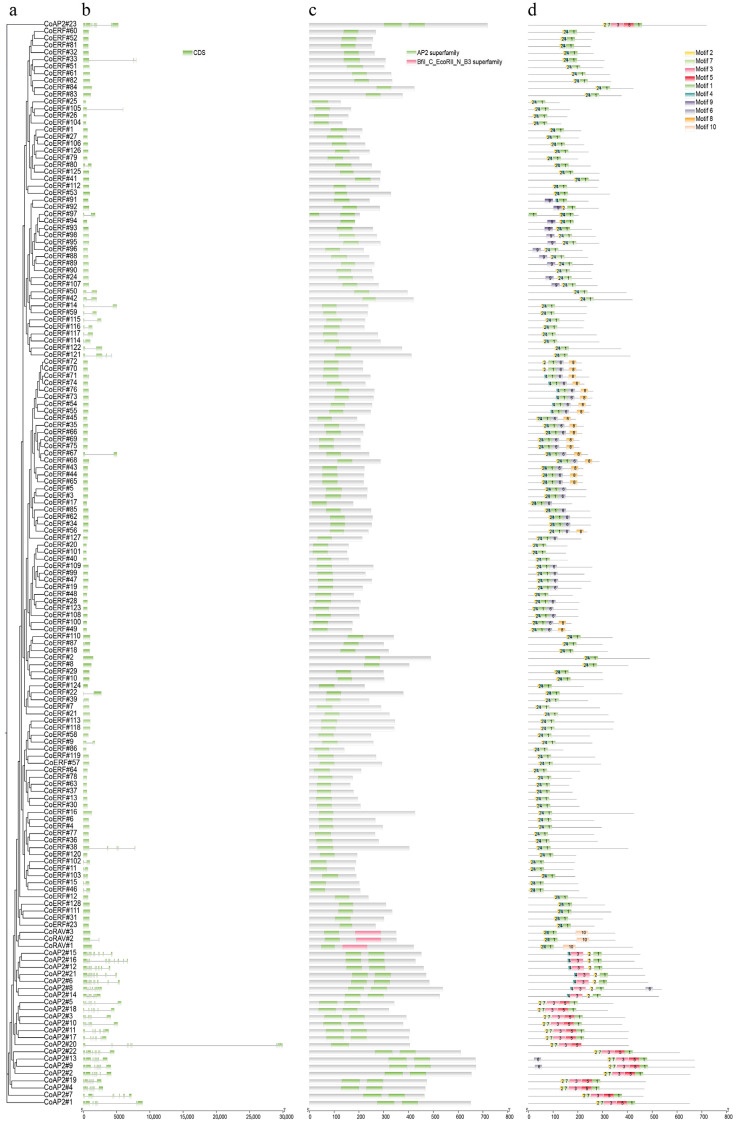
Gene structure, conserved domains and motifs analysis of AP2/ERF genes from camphor tree. (**a**) Phylogenetic classification of all the AP2/ERF genes. (**b**) Gene structure analysis as indicated by intro-exon distribution on each AP2/ERF gene. (**c**) Conserved domain analysis of all the AP2/ERF genes. Green indicates the conserved AP2/ERF domain and pink indicates the B3 domain. (**d**) Motif analysis of all the AP2/ERF genes. 10 motifs in different colors were illustrated.

Further investigation of gene structure showed that genes located in close proximity on the phylogenetic tree tend to have similar intron–exon patterns. Specifically, most of the genes from the ERF and RAV subfamilies contained only one exon, while the genes from the AP2 subfamily contained an average of nine exons (Figure 3a,b). Domain analysis revealed that the AP2/ERF domain is conserved among family members, with each gene containing one to two AP2/ERF domains. Uniquely, RAV genes contained a B3 domain, which ranged from 100 to 120 amino acids in size (Figure 3c). MEME motif analysis was conducted to identify conserved sequences that may be crucial for the specialized functions of each subfamily. Of the 10 identified motifs, motifs 1, 2, and 4 were observed in the majority of the ERF subfamily and in all three RAV genes, while motifs 8 and 9 were exclusively identified in the ERF subfamily, and motif 10 was found only in the RAV genes. In the AP2 subfamily, genes either possess the motif combination 1, 2, 3, 4, or the combination 1, 2, 3, 5, 7. Notably, the first domain of *CoERF#97*, the only two-domain ERF gene, contained only motif 1, indicating a potentially incomplete function of this domain (Figure 3d; Supplementary Table S2).

### 2.3. Identification of Cis-Acting Regulatory Elements in CoAP2/ERF Promoters

**Figure 4:**
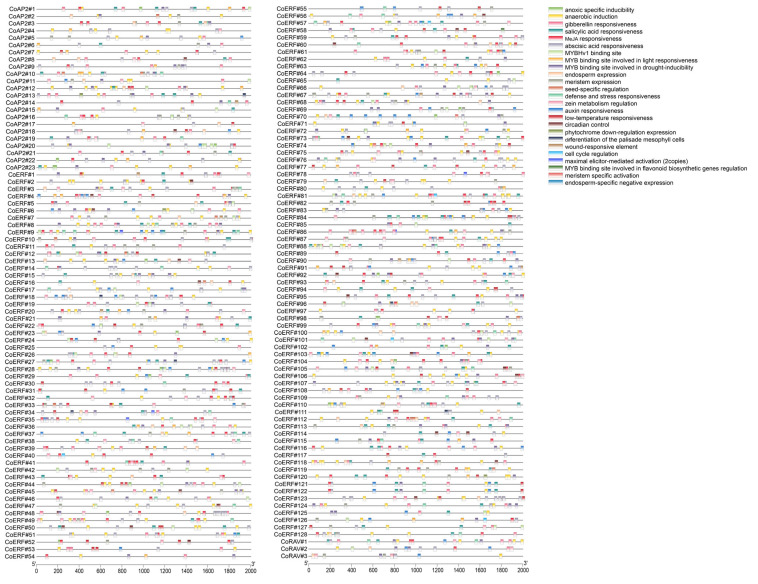
PlantCARE analysis of cis-acting regulatory elements of promoter regions of CoAP2/ERF family genes.

To enhance the understanding of the regulatory mechanisms governing CoAP2/ERF genes in response to internal and external signals, promoter regions (2 kb upstream of the start codon) of the 147 genes were isolated and analyzed using PlantCARE. This analysis identified 4041 motifs with known functions. Among them, light responsiveness motifs were identified in every single AP2/ERF family gene, therefore was not displayed in Figure 4. In addition, hormone response elements related to abscisic acid, auxin, gibberellin, MeJA, and salicylic acid were found in members across the three subfamilies. Motifs involved in plant growth regulation included those related to cell cycle regulation, circadian control, differentiation of the palisade mesophyll cells, endosperm expression, endosperm-specific negative expression, meristem expression, meristem specific activation, seed-specific regulation, and zein metabolism regulation. Additionally, motifs involved in potential biotic and abiotic responses were identified, such as those related to anaerobic induction, anoxic specific inducibility, defense and stress responsiveness, low-temperature responsiveness, maximal elicitor-mediated activation, phytochrome down-regulation expression, and wound responsiveness. Furthermore, MYB transcription factor binding sites were identified, including the MYBHv1 binding site and MYB binding sites involved in drought inducibility, flavonoid biosynthetic genes regulation, and light responsiveness. These motifs were uniquely distributed across the three subfamilies, suggesting that different sets of AP2/ERF genes might respond to plant growth and stress signals. Based on the presence of functional motifs in each subfamily, it was predicted that ERF genes are more actively involved in a broader range of biological activities, while RAV genes primarily respond to stress factors.

### 2.4. Collinearity Analysis of CoAP2/ERF Family Genes

**Figure 5:**
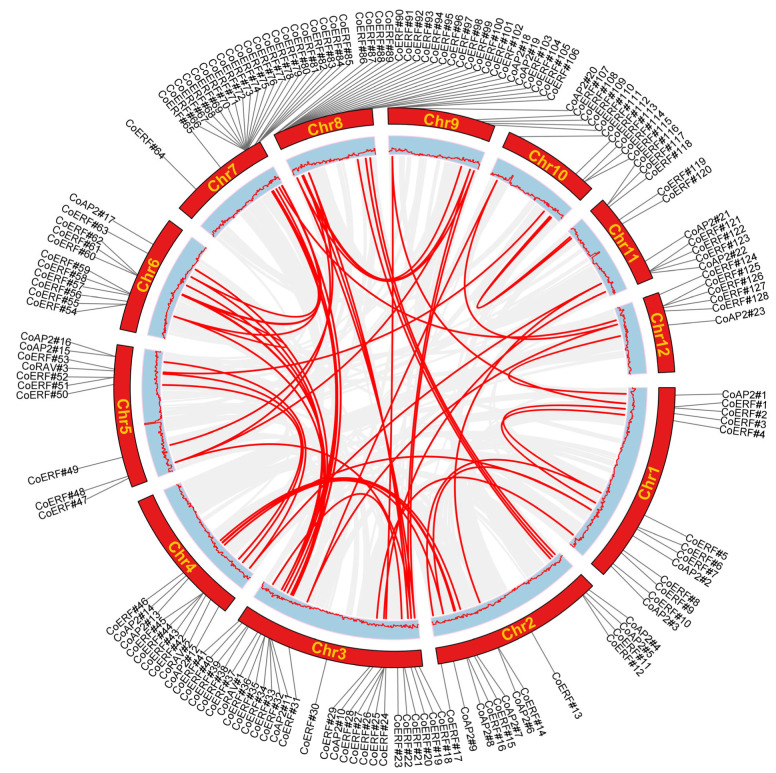
Collinearity analysis of CoAP2/ERF genes. Collinear gene pairs were linked using red lines.

To gain deeper insights into the evolutionary dynamics and functional diversification of CoAP2/ERF family genes, comprehensive collinearity analysis was performed. Our analysis identified 12 tandem duplication gene clusters among the CoAP2/ERF family members. These clusters were predominantly located on chromosomes 7 and 8, suggesting that frequent tandem duplication events occurred in these regions. Additionally, ERF genes exhibited a higher prevalence of tandem duplication compared to the other two subfamilies, indicating the existence of a potential adaptive mechanism to fine-tune specific developmental or stress signals (Supplementary Table S3). In addition to tandem duplications, 64 collinear gene pairs were identified within the family, indicating that multiple gene duplication events might have contributed to the expansion and diversification of the AP2/ERF gene family in camphor trees (Figure 5).

### 2.5. Expression Profiling of CoAP2/ERF Genes During Plant Development

**Figure 6:**
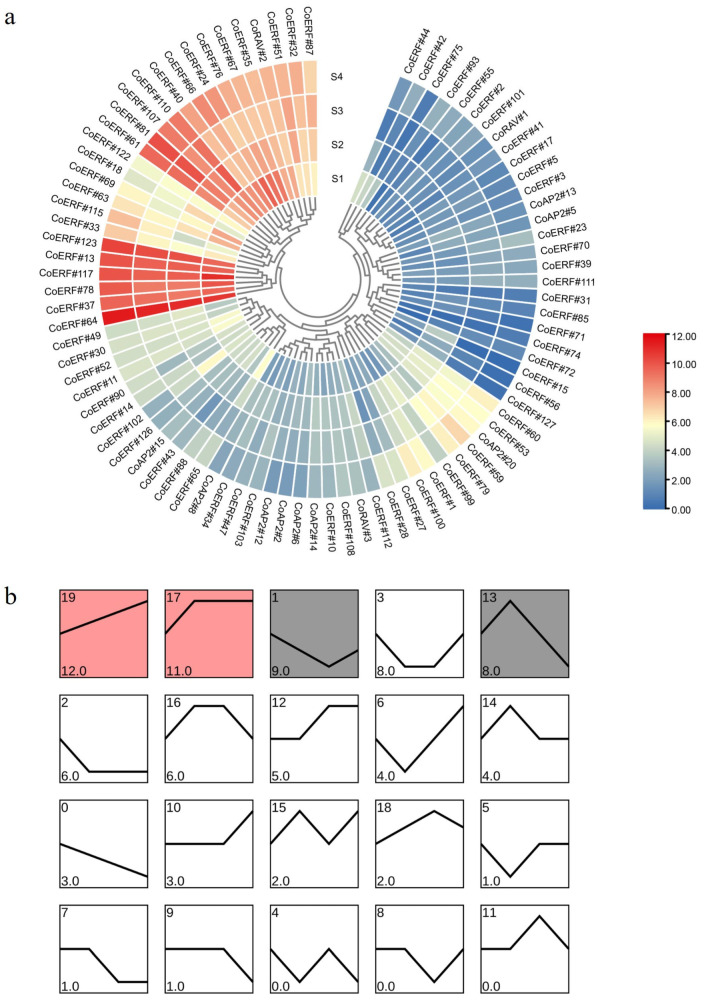
Expression profiling of expressed CoAP2/ERF genes during stem development. (**a**) Expression heatmap of expressed CoAP2/ERF genes during stem development. Expression levels were indicated by logFPKM. (**b**) Trend analysis of CoAP2/ERF genes expressed during stem development. Trend profiles were ordered according to the number of genes assigned (**bottom left**). Colored trends indicate trends with significant differences (*p* < 0.05). The number of trends was set to 20.

To gain insights into the functions of CoAP2/ERF family genes during plant development, our most recently obtained RNA-Seq data of camphor tree stems at four developmental stages (S1–S4) was utilized to detect their expression. Eighty-six out of the 147 family genes were expressed in all four developmental stages, including 74, 9, and 3 genes from ERF, AP2, and RAV subfamilies, respectively, accounting for 60.2%, 39.1%, and 100% of the total gene numbers in each corresponding subfamily. The majority of these genes exhibited consistent expression across the four developmental stages, with the expression of 16 ERF genes (*CoERF#13*, *24*, *35*, *37*, *40*, *61*, *64*, *66*, *67*, *76*, *78*, *81*, *107*, *110*, *117*, *123*) and one RAV gene (*CoRAV#2*) being significantly high throughout all stages (FPKM > 100 in all samples; Figure 6a). Further trend analysis of expressed CoAP2/ERF genes during stem development identified 17 trend blocks, with significantly enriched trends #19, #17, #1, and #13 harboring the most number of genes (Figure 6b). Of the highly expressed genes, *CoERF#64* and *CoERF#81* belonged to trend #19, *CoERF#61* and *CoERF#123* belonged to trend #17, while *CoERF#24*, *CoERF#35*, *CoERF#67*, and *CoERF#76* belonged to trend #1 (Supplementary Table S4).

### 2.6. Expression Profiling of CoAP2/ERF Genes Under E. poaceicola Infection

**Figure 7:**
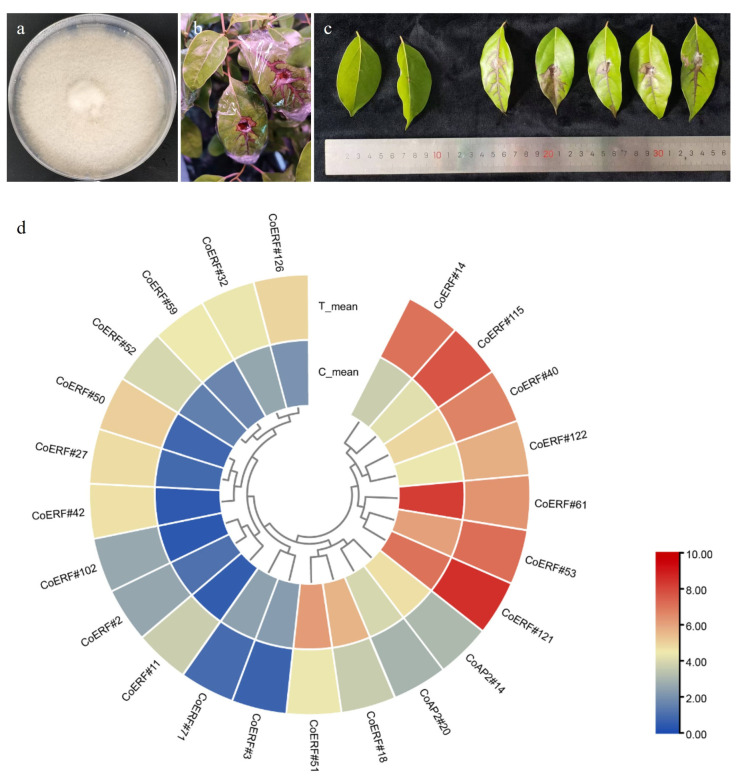
Infection of camphor tree leaves with *E. poaceicola*. (**a**) Morphology of *E. poaceicola* on PDA medium. (**b**) Symptoms of camphor tree leaves infected with *E. poaceicola*. (**c**) Comparison between control wounded leaves inoculated with PDA plugs (left two leaves) and treatment wounded leaves inoculated with *E. poaceicola* containing PDA plugs (right five leaves). (**d**) Heatmap displaying differentially expressed genes between control and treatment leaves following *E. poaceicola* infection. Expression levels are indicated by logFPKM.

As an economically important tree, *C. officinarum* is cultivated as bushes and harvested for leaves used in essential oil extraction; consequently, leaf infections can directly affect yield, and leaf spot symptoms are frequently observed. In our previous work, *E. poaceicola* was isolated from symptomatic leaves and identified as the primary causal agent of the observed disease [3]. To explore whether AP2/ERF genes respond during infection, we inoculated camphor tree leaves and sampled at the first clearly symptomatic stage (Figure 7a–c), providing a single-time-point snapshot of the transcriptional response.

RNA-seq of infected versus mock leaves identified 2842 differentially expressed genes (DEGs), including 1556 up-regulated and 1286 down-regulated transcripts (Supplementary Table S5). KEGG classification indicated broad effects on cellular metabolism, with most DEGs annotated to metabolic pathways (KO01100) and biosynthesis of secondary metabolites (KO01110). Among the DEGs, 23 genes were annotated as AP2/ERF family members; 16 were up-regulated and 7 were down-regulated in infected leaves (Figure 7d; Supplementary Table S6). Notably, all up-regulated family members belonged to the ERF subfamily (e.g., *CoERF#2*, *11*, *14*, *27*, *32*, *40*, *42*, *50*, *52*, *53*, *59*, *102*, *115*, *121*, *122*, and *126*), supporting a role for ERFs in defense-associated transcription in camphor tree. Among these, *CoERF#115* and *CoERF#121* (annotated as *RAP2.3*- and *RAP2.2-like*, respectively) showed the highest expression levels (FPKM > 200) in infected tissue, whereas *CoAP2#14*, *CoAP2#20*, *CoERF#3*, *18*, *51*, *61*, and *71* decreased upon infection. Of note, *CoERF#51* and *CoERF#61* (both *ERF5-like*) exhibited the highest expression in uninfected leaves but dropped markedly after infection (|log2FC| > 1.8). Promoter inspection indicated that these genes harbor one or more pathogen-related cis-elements, consistent with a regulatory role in the response to *E. poaceicola* (Supplementary Table S7).

### 2.7. qPCR Validation of RNA-Seq Analysis

**Figure 8:**
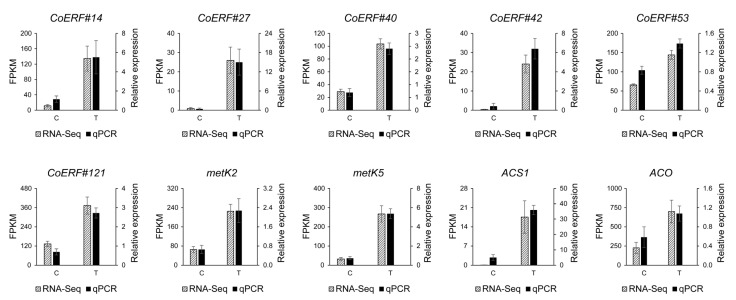
qPCR validation of RNA-Seq data. Gene expression levels were indicated by FPKM values (left y-axis) in the RNA-Seq analysis and calculated as 2^−ΔΔCT^ value (right y-axis) in the qPCR analysis.

To validate our RNA-Seq data, qPCR analysis was conducted on selected CoAP2/ERF genes that were differentially expressed in response to *E. poaceicola* infection, with relatively high expression levels (FPKM > 50). The genes analyzed included *CoERF#14*, *27*, *40*, *42*, *53*, and *121*. Consistent with our RNA-Seq data, the expression levels of these genes increased significantly in the affected leaves, confirming the reliability of our transcriptome data. Given that all the upregulated genes upon infection belonged to the ERF subfamily, also known as the Ethylene Responsive Factor subfamily, it is reasonable to assume that ethylene biosynthesis was upregulated in response to pathogen infection. Consequently, we analyzed the expression levels of genes encoding metK2 and 5 (S-adenosylmethionine synthase), ACS1 (1-aminocyclopropane-1-carboxylate synthase 1), and ACO (aminocyclopropanecarboxylate oxidase) from the “Cysteine and methionine metabolism” pathway. According to the KEGG reference pathway, metK, ACS, and ACO are the last three functional enzymes in ethylene production. The upregulation of these enzyme-encoding genes suggests an increase in ethylene formation upon infection. This upregulation was consistently observed in both our RNA-Seq and qPCR analyses (Figure 8).

## 3. Discussion

Transcription factors are specialized proteins that contain one or more specific DNA-binding domains, that could recognize specific DNA sequences, therefore regulating gene expressions [25]. The AP2/ERF family is a vital plant transcription factor family function in plant biological and physiological processes like responses to stress and defense, fundamental growth and development, and metabolism [6]. However, the functions of this gene family in camphor trees were barely investigated.

Our study identified 154 AP2/ERF genes, with more than 80% belonging to the ERF subfamily (Table 1). This finding is consistent with trends observed in angiosperms, where the ERF subfamily is typically the largest. However, the number of AP2/ERF genes does not directly correlate with genome size. For example, the genome size of camphor tree is approximately 750 Mb, yet 147 and 191 AP2/ERF genes were identified in Arabidopsis (125 Mb) and pear (*Pyrus bretschneideri*; 527 Mb), respectively [7,26]. This suggests that in addition to genome size, certain selective pressures and functional diversification may also drive the expansion of the AP2/ERF gene family across different plant lineages. Moreover, lineage-specific gene loss or divergence could be inferred by the presence of the Soloist subfamily members in model species like Arabidopsis but their absence in camphor trees [27,28,29].

Tandem duplication of CoAP2/ERF gene clusters was intensively observed on chromosomes 7 and 8, implying that these regions might serve as hotspots for genetic variation. Additionally, the identification of collinear gene pairs suggests that segmental duplications also significantly contributed to family diversification, enabling functional redundancy or neofunctionalization (Figure 5; Supplementary Table S3). Considering the broad diversity of colonizing niches of angiosperms, and the general trade-off between plant growth and defense mechanisms, the emergence of AP2/ERF gene paralogs over evolutionary course could facilitate more precise regulation of stress response while minimizing possible impacts of these stress-responsive genes on plant growth. As observed in Arabidopsis, although constitutive overexpression of *AtCBF1–4* enhanced plant’s tolerance to freezing and drought stresses, expression of different sets of *AtCBF* genes were induced, with *AtCBF1–3* induced under cold stress and *AtCBF4* induced under drought stress [29].

Gene structure analysis revealed that, with the exception of three small clades in the ERF subfamily, CoERF/RAV genes generally exhibited a single-exon structure, whereas all the CoAP2 genes harbored multi-exon structures. This observation indicated distinct evolutionary trajectories between these subfamilies. Since multi-exon genes are often associated with a higher potential for generating diverse transcripts through alternative splicing, these multi-exon-containing CoAP2/ERF genes are more inclined to have varied or tissue-specific functions. As demonstrated in *Andrographis paniculata*, the expression levels of isoforms of different genes altered differently upon MeJA treatment, suggesting that alternative splicing events under MeJA treatment could act as regulatory mechanisms in controlling plant growth and responses to stresses [30]. Furthermore, in addition to the conserved AP2/ERF domain, subfamily-specific domains (e.g., B3 domain in RAV) were also identified, implying functional specialization within the gene family [31]. This could be more clearly illustrated by motif analysis, which identified motifs 3, 5, and 7 exclusively in the AP2 subfamily, motifs 8 and 9 in ERF, and motif 10 in RAV (Figure 3). In contrast, motifs 1 and 2 (except for seven ERF genes) were conserved across subfamilies, corresponding to the RAYD and YRG elements, accordingly (Supplementary Table S2). The YRG element is well known for facilitating DNA binding, while the RAYD element enhances structural stability and mediates protein–protein interactions [32]. Therefore, conserved presence of these two motifs in CoAP2/ERF genes underlines their critical roles in regulatory activity, whereas variation in motif combinations, even within the same subfamily, illustrates functional divergence (e.g., developmental versus stress-defense roles).

Promoter analysis identified a diverse array of hormone- and stress- responsive elements, linking CoAP2/ERF genes to hormonal signaling pathways, such as abscisic acid, auxin, and gibberellin, as well as stress responses (Figure 4). Through expression profiling of CoAP2/ERF genes during stem development, it was revealed that approximately 60% of these genes are constitutively expressed. Notably, *CoERF#64* and *CoERF#81* in Trend #19, along with *CoERF#61* and *CoERF#123* in Trend #17, showed consistently high expression levels across all developmental stages. This observation suggested the involvement of these genes in maintaining basal cellular functions. In contrast, trend analysis identified stage-specific expression modules, such as Trend #13, indicating that subgroups of CoAP2/ERF genes may play roles in fine-tuning developmental transitions, including lignification or vascular formation, which are critical for secondary growth of woody stems (Figure 6). The regulatory role of CoAP2/ERF genes in secondary cell wall metabolism has long been suggested in plants such as Arabidopsis and aspen [8,33].

AP2/ERF genes also play significant roles in pathogen defense. Our analysis identified 23 DEGs in the leaves following *E. poaceicola* infection (Figure 7; Supplementary Table S6). In particular, the strongly induced expression of *CoERF#115* and *CoERF#121*, orthologs of *RAP2.2/2.3*, was consistent with their well-established functions. *RAP2.2/2.3-like* genes are known to directly activate defense by activating GCC-box-containing defense genes, such as *PDF1.2/defensin-like*, *PR3/PR4 chitinases*, and *β-1,3-glucanases*. In addition, they can mediate plant responses to infection-induced low-oxygen niches by activating hypoxia/fermentation genes as well as ROS/homeostasis enzymes-encoding genes [34,35]. By contrast, seven genes, including two AP2 and five ERF genes, constitutively expressed in the stems, were downregulated upon infection. This downregulation indicated a suppression of developmental pathways in favor of prioritizing defense, possibly by fine-tuning the intensity and timing of jasmonic acid/ethylene outputs-a phenomenon commonly recognized as the growth–defense trade-off [36,37].

However, the specific roles of individual AP2/ERF genes require further functional validation, as focusing solely on stem development limits insights into the tissue-specific functions of this gene family. Moreover, given the potential for species- or context-specific functional divergence, studying a single pathogen model could not capture broader stress adaptation mechanisms. For instance, our analysis showed that the expression levels of two *ERF5* homologs, *CoERF#51* and *CoERF#61*, decreased dramatically following *E. poaceicola* infection, supporting their negative roles in fungal defense. In contrast, studies in Arabidopsis reveal functional plasticity in the *ERF5* homolog (*AtERF5*), which negatively regulates defense against a fungal pathogen but positively regulates defense against a bacterial pathogen by differentially modulating chitin signaling and other defense pathways [38].

While our RNA-seq and promoter surveys provide expression-level evidence, the mode of action of specific *C*oAP2/ERFs can be further contextualized by orthology to well-characterized Arabidopsis and poplar homologs. On the defense side, ERF proteins that operate in the JA/ET “ERF-branch” (EIN3/EIL1→ORA59/ERF1 axis) typically bind GCC-box elements to induce antifungal effectors, including PDF1.2/defensins, PR3/PR4 chitinases, and β-1,3-glucanases, together with enzymes modulating ROS and cell-wall fortification (peroxidases, callose synthases, PGIP) [34,35]. The strong induction of *CoERF#115* and *CoERF#121*—annotated as *RAP2.3/2.2-like* (ERF-VII)—is consistent with the oxygen-sensing N-end-rule pathway that stabilizes ERF-VIIs under local hypoxia at infection sites, thereby up-regulating fermentation/hypoxia genes (e.g., *ADH1*, *PDC1*) and ROS-homeostasis components (e.g., peroxidases, GSTs). Given that fungal PAMPs (e.g., chitin) primarily engage CERK1/LYK5–MPK3/6–WRKY33 signaling before converging on the ERF branch, we infer that many ERF-upregulated targets during *E. poaceicola* challenge are fungal-leaning (GCC-box program) rather than strictly broad-spectrum/SA-dominant outputs (e.g., PR1). By contrast, the marked decrease in *CoERF#51/#61* (*ERF5-like*)—which shows context-dependent roles against fungi vs. bacteria in Arabidopsis—may reflect pathogen-type-specific rewiring (e.g., dampening of *ERF5*-associated branches that are beneficial in bacterial contexts but less so under necrotrophic fungal pressure) [38]. These propositions are orthology-guided hypotheses that can be tested by examining GCC-box enrichment in DEGs and by tracking JA/ET (PDF1.2) vs. SA (PR1) marker expression over a time course.

On the developmental side, several highly expressed *C*oERFs across stem stages likely intersect with the secondary-cell-wall (SCW) regulatory hierarchy: NAC master regulators (SND1/NST1, VNDs) upstream of MYB46/83, culminating in deposition programs involving cellulose synthases (*CesA4/7/8*) and lignin biosynthetic genes (*PAL*, *C4H*, *4CL*, *HCT*, *C3H*, *CCoAOMT*, *COMT*, *CCR*, *CAD*) and *laccases*. ERF subgroups (e.g., *ERF071*/*WIND-like* and *ERF018*/*109-like*) have been implicated in cambial activity, wound signaling, and cell-wall remodeling, offering a mechanistic route for *C*oERFs that show constitutive or stage-biased expression in stems to influence vascular differentiation, lignification, and mechanical strengthening (Figure 6). RAV members, by virtue of the B3 domain, may further integrate BR/ABA/ethylene crosstalk, fine-tuning growth–defense trade-offs at developmental checkpoints.

Despite these complexities, the comprehensive analysis of AP2/ERF transcription factor family in camphor tree offers critical insights into their evolutionary dynamics, structural diversity, and functional specialization. Additionally, expression profiling across stem development and under *E. poaceicola* infection offers a foundational understanding of their roles in the development and stress resilience of camphor trees.

## 4. Materials and Methods

### 4.1. Plant Materials and Infection Assay

Three-year-old host plants of camphor trees (voucher number L3-12) were grown in the experimental garden of the Guangdong Academy of Forestry. Three healthy plants with identical growth conditions were selected for infection treatment with *E. poaceicola*. *E. poaceicola* was streaked onto the surface of solidified Potato Dextrose Agar (PDA; Solarbio, Beijing, China) plates using a sterile loop and incubated at 25 °C for 3 days to promote sporulation.

Infection assays involved wounding the upper side of the leaf surface with a sterilized insect needle, followed by the application of a PDA plug containing *E. poaceicola* spores, which was then wrapped with plastic wrap. Control plants were subjected to the same treatment but with an empty PDA plug. After inoculation, the plants were maintained in a high-humid environment (70%) for three days to facilitate fungal penetration and infection. Infected and uninfected leaves were collected and snap-frozen in liquid nitrogen prior to RNA sequencing and qPCR analysis.

### 4.2. Genome-Wide Identification of CoAP2/ERF Genes

Genome sequences of camphor tree were downloaded from the NCBI BioProject database under accession number PRJNA1000241 [39]. Hidden Markov Model profile of the AP2/ERF domain (PF00847) procured from the Pfam protein family dataset (https://ebi.ac.uk/interpro/; accessed on 5 December 2024) was used for the sequence blast against the sequenced genome with set E-value cutoff of 0.001. Obtained sequences were subjected to online NCBI Conserved Domains (https://www.ncbi.nlm.nih.gov/cdd; accessed on 5 December 2024) and Pfam (https://ebi.ac.uk/interpro/; accessed on 5 December 2024) databases to confirm the presence of conserved motifs. Genes without a conserved domain were removed from the list.

### 4.3. Phylogenetic Analysis and Classification of the CoAP2/ERF Genes

For the phylogenetic analysis of AP2/ERF family genes identified in camphor tree and Arabidopsis, confirmed genes were first aligned using ClustalW software built in MEGA 11 with default parameters. Prior to tree construction, divergent or poorly aligned regions were manually deleted. Phylogenetic trees were then constructed using the Maximum Likelihood (ML) approach with a bootstrap test of 1000 replicates. The CoAP2/ERF family members were then classified according to the phylogenetic construction result and the classification scheme proposed by Nakano et al. [7].

### 4.4. Chromosomal Mapping and Collinearity Analyses

CoAP2/ERF family members were mapped to the camphor tree genome using TBtools (v. 2.332) [40]. The genes were then renamed according to their subclass, as determined by phylogenetic classification, and numbered sequentially based on their chromosomal locations. Gene duplications were investigated using multiple collinear scanning toolkits (MCScanX) and syntenic mapping was depicted using the Dual Synteny Plotter software built in TBtools.

### 4.5. Gene Structure Analysis, Motif Analysis, and Prediction of Cis-Acting Regulatory Elements

Gene structure of CoAP2/ERF family members was analyzed using the online software GSDS 2.0 [41]. Conserved motifs within this gene family were identified using MEME [42] and visualized using TBtools. Initially, 20 conserved motifs were selected and only those characteristics of the four subfamilies were displayed. Cis-acting regulatory elements of promoter regions of these genes were predicted using PlantCARE database [43].

### 4.6. RNA Sequencing

mRNA was extracted and sent to Gene Denovo Biotechnology Co. (Guangzhou, China) for sequencing. The extracted mRNA underwent enrichment with mRNA Capture Beads. After purification, the mRNA was subjected to high temperatures to induce fragmentation. The fragmented mRNA was then utilized as template for cDNA synthesis. After adapter ligation, Hieff NGS^®^ DNA Selection Beads were used to purify and select target fragments. Following PCR library amplification, the Illumina Novaseq X Plus system was applied for sequence detection. Lastly, raw reads were filtered by removing adapter sequences and low-quality reads, resulting in over 36.5 million clean reads per sample in our infection experiment. The same reference genome used for the identification of CoAP2/ERF family genes was employed for sequence alignment. Raw sequencing reads were submitted to NCBI (project number: PRJNA1280518). 

### 4.7. Expression Analysis of CoAP2/ERF Genes During Plant Development and Under Pathogen Infection

Sequencing data from developing stems at four developmental stages of borneol-type camphor trees were obtained from our most recent study (data submitted to NCBI, project number: PRJNA1198615). In that study, expression profiles of developing stems from four developmental stages of linalool- and borneol-type camphor trees were generated to investigate the drivers of chemotypic differences in the developing stems of camphor trees (manuscript submitted; under review). Expression levels of CoAP2/ERF genes were calculated as FPKM, and their expression heatmap was constructed using TBtools. Trend analysis was conducted using Stem [44].

Similarly, gene expression levels in the infection treatment experiment were also calculated as FPKM. Genes with a fold change ≥2 and a *p*-value < 0.01 between the infected and uninfected groups were identified as differentially expressed genes (DEGs). CoAP2/ERF genes reactive to *E. poaceicola* infection were then cross-checked with constitutively expressed CoAP2/ERF genes.

### 4.8. qPCR Validation of RNA-Seq Data

qPCR was performed to validate our RNA-seq data. In this study, a total of ten CoAP2/ERF and ethylene biosynthesis genes were selected for validation. Primers were designed using Primer Premier 5 according to sequences obtained by annotation. Primer sequences were listed in Supplementary Table S8.

## 5. Conclusions

This study provides the first genome-wide characterization of the AP2/ERF gene family in camphor trees, revealing lineage-specific expansion of the family through tandem and segmental duplications. Their integration into ethylene-mediated defense signaling offers novel insight into how gene family expansion contributes to ecological adaptation in perennial tree species. Moreover, our expression profiling illustrates that CoAP2/ERF genes play dual roles in developmental transitions and pathogen defense. Notably, the dramatically upregulated expression of *RAP2.2* and *RAP2.3* homologs upon *E. poaceicola* infection supported the roles of ERF genes in hypoxia and antimicrobial responses. Conversely, dramatic downregulation of the constitutively expressed *ERF5* homologs indicated a potential growth–defense trade-off in camphor trees. These findings not only advance our understanding of transcriptional regulation in *C. camphora* but also establish a valuable genetic framework for improving tree resilience through molecular breeding. Further investigations should focus on functional validation of candidate CoAP2/ERF genes using gene editing approaches such as CRISPR/Cas and VIGS, alongside integrative multi-omics analyses.

## Figures and Tables

**Table 1:** Classification of CoAP2/ERF genes.

Classification	Characteristics	No.
AP2 subfamily	one AP2/ERF domain	1
	two AP2/ERF domains	22
ERF subfamily	one AP2/ERF domain	127
	two AP2/ERF domains	1
RAV subfamily	B3 domain	3
	Total	154

## Data Availability

All data generated or analyzed during this study are included in this published article and its Supplementary Materials .

## Supplementary Materials

The following supporting information can be downloaded at https://www.mdpi.com/article/10.3390/plants14172694/s1, Table S1: Classification and re-naming of CoAP2/ERF genes; Table S2: Conserved sequences of identified motifs and the number of each motif in subfamilies; Table S3: Collinear gene pairs; Table S4: Trend analysis of CoAP2/ERF genes; Table S5: Differentially expressed genes between uninfected and *E. poaceicola*-infected camphor tree leaves; Table S6: Differentially expressed genes belonging to the AP2/ERF gene family; Table S7: Potential pathogen defense-related cis-acting regulatory elements in differentially expressed CoAP2/ERF genes upon *E. poaceicola* infection; Table S8: qPCR primer sequence list.

## References

[1] Hou J., Zhang J., Zhang B., Jin X., Zhang H., Jin Z. (2020). Transcriptional analysis of metabolic pathways and regulatory mechanisms of essential oil biosynthesis in the leaves of *Cinnamomum camphora* (L.) Presl. Front. Genet..

[2] Chen J., Wu F., Wu Z., Yu Z., Yang Y., Ma H., Wu J. (2023). First report of ‘*Candidatus* Phytoplasma asteris’ associated with witches’-broom disease of *Cinnamomum camphora* in China. Plant Dis..

[3] Li D., Zhang T., Song Q., Liu J., Zhang H., Luan F. (2022). First report of leaf spot disease on *Cinnamomum camphora* (Camphor Tree) caused by *Epicoccum poaceicola* in China. Plant Dis..

[4] Liu H., Li D., Zhang T., Zhang H., Song Q., Liu J., Yang Q., Luan F., Li D. (2022). First Report of Anthracnose on *Cinnamomum camphora* (Camphor tree) Caused by *Colletotrichum fioriniae* and *Colletotrichum siamense* in China. Plant Dis..

[5] Xiao Y., Li M., Chen F. (2023). Root rot of *Cinnamomum camphora* (Linn) presl caused by *phytopythium vexans* in China. Plants.

[6] Feng K., Hou X., Xing G., Liu J., Duan A., Xu Z., Li M., Zhuang J., Xiong A. (2020). Advances in AP2/ERF super-family transcription factors in plant. Crit. Rev. Biotechnol..

[7] Nakano T., Suzuki K., Fujimura T., Shinshi H. (2006). Genome-wide analysis of the ERF gene family in *Arabidopsis* and rice. Plant Physiol..

[8] van Raemdonck D., Pesquet E., Cloquet S., Beeckman H., Boerjan W., Goffner D., El Jaziri M., Baucher M. (2005). Molecular changes associated with the setting up of secondary growth in aspen. J. Exp. Bot..

[9] Xing H., Jiang Y., Zou Y., Long X., Wu X., Ren Y., Li Y., Li H. (2021). Genome-wide investigation of the AP2/ERF gene family in ginger: Evolution and expression profiling during development and abiotic stresses. BMC Plant Biol..

[10] Yang H., Sun Y., Wang H., Zhao T., Xu X., Jiang J., Li J. (2021). Genome-wide identification and functional analysis of the ERF2 gene family in response to disease resistance against *Stemphylium lycopersici* in tomato. BMC Plant Biol..

[11] Zhuang J., Peng R., Cheng Z., Zhang J., Cai B., Zhang Z., Gao F., Zhu B., Fu X., Jin X. (2009). Genome-wide analysis of the putative AP2/ERF family genes in *Vitis vinifera*. Sci. Hortic..

[12] Riechmann J.L., Heard J., Martin G., Reuber L., Jiang C., Keddie J., Adam L., Pineda O., Ratcliffe O.J., Samaha R.R. (2000). Arabidopsis transcription factors: Genome-wide comparative analysis among eukaryotes. Science.

[13] Sakuma Y., Liu Q., Dubouzet J.G., Abe H., Shinozaki K., Yamaguchi-Shinozaki K. (2002). DNA-Binding specificity of the ERF/AP2 domain of *Arabidopsis* DREBs, transcription factors involved in dehydration- and cold-inducible gene expression. Biochem. Biophys. Res. Commun..

[14] Aukerman M.J., Sakai H. (2003). Regulation of flowering time and floral organ identity by a MicroRNA and its APETALA2-like target genes. Plant Cell.

[15] Luo C., Wang S., Ning K., Chen Z., Wang Y., Yang J., Qi M., Wang Q. (2021). The APETALA2 transcription factor LsAP2 regulates seed shape in lettuce. J. Exp. Bot..

[16] Mehrnia M., Balazadeh S., Zanor M.I., Mueller-Roeber B. (2013). EBE, an AP2/ERF transcription factor highly expressed in proliferating cells, affects shoot architecture in *Arabidopsis*. Plant Physiol..

[17] Baillo E.H., Kimotho R.N., Zhang Z., Xu P. (2019). Transcription factors associated with abiotic and biotic stress tolerance and their potential for crops improvement. Genes.

[18] Mizoi J., Shinozaki K., Yamaguchi-Shinozaki K. (2012). AP2/ERF family transcription factors in plant abiotic stress responses. Biochim. Biophys. Acta.

[19] Shoji T., Yuan L. (2021). ERF gene clusters: Working together to regulate metabolism. Trends Plant Sci..

[20] Song J., Chen C., Zhang S., Wang J., Huang Z., Chen M., Cao B., Zhu Z., Lei J. (2020). Systematic analysis of the Capsicum ERF transcription factor family: Identification of regulatory factors involved in the regulation of species-specific metabolites. BMC Genom..

[21] Müller M., Munné-Bosch S. (2015). Ethylene response factors: A key regulatory hub in hormone and stress signaling. Plant Physiol..

[22] Chandan R.K., Kumar R., Swain D.M., Ghosh S., Bhagat P.K., Patel S., Bagler G., Sinha A.K., Jha G. (2023). RAV1 family members function as transcriptional regulators and play a positive role in plant disease resistance. Plant J..

[23] Woo H.R., Kim J.H., Kim J., Kim J., Lee U., Song I.J., Kim J.H., Lee H.Y., Nam H.G., Lim P.O. (2010). The RAV1 transcription factor positively regulates leaf senescence in *Arabidopsis*. J. Exp. Bot..

[24] Qin Z., Chen C., Zhang T., Wu Y., Zheng Y. (2025). Single-cell RNA sequencing reveals transcriptional regulation and metabolic pathways of terpenoid biosynthesis in developing *Cinnamomum camphora* leaf cells. Curr. Plant Biol..

[25] Babu M.M., Luscombe N.M., Aravind L., Gerstein M., Teichmann S.A. (2004). Structure and evolution of transcriptional regulatory networks. Curr. Opin. Struct. Biol..

[26] Li X., Tao S., Wei S., Ming M., Huang X., Zhang S., Wu J. (2018). The mining and evolutionary investigation of AP2/ERF genes in pear (*Pyrus*). BMC Plant Biol..

[27] Han J., Xie X., Zhang Y., Yu X., He G., Li Y., Yang G. (2022). Evolution of the DEHYDRATION-RESPONSIVE ELEMENT-BINDING PROTEIN subfamily in green plants. Plant Physiol..

[28] Kerstens M.H., Schranz M.E., Bouwmeester K. (2020). Phylogenomic analysis of the APETALA2 transcription factor subfamily across angiosperms reveals both deep conservation and lineage-specific patterns. Plant J..

[29] Wang L., Ma H., Lin J. (2019). Angiosperm-wide and family-level analyses of AP2/ERF genes reveal differential retention and sequence divergence after whole-genome duplication. Front. Plant Sci..

[30] Yao W., An T., Xu Z., Zhang L., Gao H., Sun W., Liao B., Jiang C., Liu Z., Duan L. (2020). Genomic-wide identification and expression analysis of AP2/ERF transcription factors related to andrographolide biosynthesis in *Andrographis paniculata*. Ind. Crops Prod..

[31] Choudhury S. (2024). Computational analysis of the AP2/ERF family in crops genome. BMC Genom..

[32] Ma Z., Hu L., Jiang W. (2024). Understanding AP2/ERF transcription factor responses and tolerance to various abiotic stresses in plants: A comprehensive review. Int. J. Mol. Sci..

[33] Lasserre E., Jobet E., Llauro C., Delseny M. (2008). AtERF38 (At2g35700), an AP2/ERF family transcription factor gene from *Arabidopsis thaliana*, is expressed in specific cell types of roots, stems and seeds that undergo suberization. Plant Physiol. Biochem..

[34] Hinz M., Wilson I.W., Yang J., Buerstenbinder K., Llewellyn D., Dennis E.S., Sauter M., Dolferus R. (2010). *Arabidopsis* RAP2.2: An ethylene response transcription factor that is important for hypoxia survival. Plant Physiol..

[35] Wamaitha M.J., Yamamoto R., Wong H.L., Kawasaki T., Kawano Y., Shimamoto K. (2012). OsRap2.6 transcription factor contributes to rice innate immunity through its interaction with Receptor for Activated Kinase-C 1 (RACK1). Rice.

[36] Xie Z., Nolan T.M., Jiang H., Yin Y. (2019). AP2/ERF transcription factor regulatory networks in hormone and abiotic stress responses in *Arabidopsis*. Front. Plant Sci..

[37] Zhou Y., Zheng R., Peng Y., Chen J., Zhu X., Xie K., Su Q., Huang R., Zhan S., Peng D. (2023). Bioinformatic assessment and expression profiles of the AP2/ERF superfamily in the *Melastoma dodecandrum* genome. Int. J. Mol. Sci..

[38] Son G.H., Wan J., Kim H.J., Nguyen X.C., Chung W.S., Hong J.C., Stacey G. (2012). Ethylene-responsive element-binding factor 5, ERF5, is involved in chitin-induced innate immunity response. Mol. Plant Microbe Interact..

[39] Li D., Lin H., Wang X., Bi B., Gao Y., Shao L., Zhang R., Liang Y., Xia Y., Zhao Y. (2023). Genome and whole-genome resequencing of *Cinnamomum camphora* elucidate its dominance in subtropical urban landscapes. BMC Biol..

[40] Chen C., Chen H., Zhang Y., Thomas H.R., Frank M.H., He Y., Xia R. (2020). TBtools: An integrative toolkit developed for interactive analyses of big biological data. Mol. Plant.

[41] Hu B., Jin J., Guo A.Y., Zhang H., Luo J., Gao G. (2015). GSDS 2.0: An upgraded gene feature visualization server. Bioinformatics.

[42] Bailey T.L., Johnson J., Grant C.E., Noble W.S. (2015). The MEME Suite. Nucleic Acids Res..

[43] Lescot M., Déhais P., Thijs G., Marchal K., Moreau Y., Van de Peer Y., Rouzé P., Rombauts S. (2002). PlantCARE, a database of plant cis-acting regulatory elements and a portal to tools for in silico analysis of promoter sequences. Nucleic Acids Res..

[44] Ernst J., Bar-Joseph Z. (2006). STEM: A tool for the analysis of short time series gene expression data. BMC Bioinform..

